# Clinical and Patient-Reported Outcomes for Intraoral (Palatal and Tuberosity) Soft Tissue Grafts in Root Coverage Procedures: A Systematic Review

**DOI:** 10.3390/dj13120563

**Published:** 2025-12-01

**Authors:** Suha Alyawar, Fatima Al Zahra, Eman Aljoghaiman, Faisal E. Aljofi, Adel S. Alagl, Marwa Madi

**Affiliations:** 1 Fellowship in Periodontics Program, Department of Preventive Dental Sciences, Division of Periodontics, College of Dentistry, Imam Abdulrahman Bin Faisal University, P.O. Box 1982, Dammam 31441, Saudi Arabia; 2220600014@iau.edu.sa (S.A.); 2220600015@iau.edu.sa (F.A.Z.); 2 Department of Preventive Dental Sciences, Division of Periodontics, College of Dentistry, Imam, Abdulrahman Bin Faisal University, P.O. Box 1982, Dammam 31441, Saudi Arabia; ealjoghaiman@iau.edu.sa (E.A.); fealjofi@iau.edu.sa (F.E.A.); aalagl@iau.edu.sa (A.S.A.)

**Keywords:** soft tissue, free gingival graft, connective tissue graft, tuberosity, hard palate

## Abstract

**Background/Objectives**: To systematically compare the clinical and patient-reported outcomes of soft tissue grafts harvested from the palate and tuberosity, in root coverage surgeries. The primary outcomes assessed were graft dimension, tissue thickness, and postoperative discomfort. **Methods**: A comprehensive search was conducted in PubMed, Web of Science, OVID Medline, and Scopus databases, covering studies published till December 2023. Eligible studies included clinical studies and clinical trials involving medically fit adults who underwent intraoral soft tissue grafting for mucogingival procedures around teeth. A total of 1209 records were initially identified, with 13 studies meeting the inclusion criteria. Data was extracted and assessed for bias. **Results**: Graft dimension in terms of thickness was generally higher for tuberosity grafts (2.9 ± 0.5 mm) compared to palatal grafts (2.3 ± 0.6 mm). Tuberosity grafts demonstrate less volume changes in buccolingual thickness. One study reported decreased postoperative pain for tuberosity compared to palate donor sites. Risk of bias assessment using ROB 2 and ROBINS-I tools showed that most included studies exhibited low risk across key domains. Among randomized trials, two studies raised some concerns due to limitations in blinding and allocation concealment. Non-randomized studies showed a moderate risk primarily in confounding and outcome measurement, consistent with inherent observational design limitations. **Conclusions**: The palate remains a well-established and reliable source of soft tissue grafts. Limited evidence from a single short-term comparative clinical study suggests that tuberosity may offer potential advantages, such as greater graft thickness, reduced volume changes, and less postoperative discomfort. However, the comparative evidence between tuberosity and palatal donor sites is derived from a single short-term study and conclusions must therefore be interpreted with caution. Standardized clinical trials with long-term follow-up are needed to confirm these observations. **Clinical Relevance**: This review provides clinicians with a preliminary evidence-based perspective into the use of tuberosity as a donor site for soft tissue grafting, an area with limited published data, and highlights its potential to enhance patient outcomes and comfort in mucogingival surgery and emphasizing the need for further research in this area.

## 1. Introduction

Soft tissue augmentation procedures are growing in popularity with a steady increase in demand [1]. Several approaches have been implemented to augment the width and thickness of keratinized tissues. However, autogenous connective tissue grafts (CTGs) continue to be regarded as the gold standard for root coverage in periodontal plastic surgery [1]. The two common sites for harvesting tissue grafts intra-orally are the hard palate, and tuberosity [2].

A variety of techniques have been proposed for harvesting connective tissue, with the goal of obtaining properties related to superior root coverage outcomes with less morbidity. Traditionally, tissues are collected from the lateral palate between the first molar and the canine [3,4,5,6]. According to Sullivan and Atkins, the utilization of grafts composed solely of lamina propria resulted in a greater augmentation of buccal gingival thickness after recession coverage. Additionally, it is plausible that the presence of fat and glandular tissue remnants could impede plasmatic circulation and revascularization during the initial stages of healing [7].

The blood supply of the oral cavity, and specifically the palate, displays a complicated pattern. The greater palatine artery (GPA) is responsible for supplying a significant portion of the hard palate. It originates from the greater palatine foramen, which is situated between the second and third maxillary molars. From there, it courses anteriorly and eventually enters the incisive foramen, where it forms a connection with the nasopalatine artery (NPA) by an anastomosis [8]. According to Reiser et al., it was shown that the greater palatine (GP) neurovascular bundle is located at 17 mm from the gingival margin in patients with a high palatal vault, 12 mm in patients with a medium palatal vault, and within 7 mm in patients with a low palatal vault [9]. A minor branch or two are sent to the tuberosity by the GPA and lesser palatal artery (LPA), which provide nourishment to this region [8]. The smaller size and lower density of GPA and LPA branches that are oriented towards the tuberosity can account for the extended process of graft revascularization and integration, as well as the frequent occurrence of necrosis in tuberosity grafts [8].

The amount of harvested tissue from the tuberosity can be limited by the presence of third molars or after periodontal surgery, and in such instances, harvesting from the lateral palate may be a more favorable decision [3]. While palatal grafts typically provide a greater surface area, their dimensional stability can be influenced by variations in the thickness and consistency of the submucosa [4,5].

It has been reported that the thickness of donor tissues obtained from the tuberosity is greater in comparison to those obtained from the palate [10]. This could be due to the nature of the tuberosity area, in which the connective tissue overlaying the tuberosity primarily consists of dense collagen fibers, with a limited amount of fatty or glandular components, covered by a well-keratinized epithelial layer, which is thinner in comparison to the palatal epithelium [3,10,11,12].

Unlike palatal harvesting, healing following tuberosity harvest as reported by Amin et al. might not be crucial to patients’ postoperative morbidity. This is due to the fact that tuberosity is subjected to less friction during mastication and does not come into direct contact with the tongue [2]. Additionally, harvesting from the tuberosity region carries a low probability of encountering intra- or postoperative complications as observed in previous studies [2,3].

Despite the widespread use of autogenous intraoral grafts for mucogingival surgeries, there is still limited evidence directly comparing outcomes associated with palate versus tuberosity donor sites. Most available studies focus on surgical techniques or short-term clinical results, with less emphasis on tissue harvest quality, patient-reported experiences, and long-term implications. This knowledge gap emphasizes the need for a systematic synthesis of the literature to clarify whether donor site selection influences both clinical outcomes and patient perspectives. Accordingly, the present systematic review aims to evaluate tissue quality and patient-reported outcomes following soft tissue grafts harvested from intraoral donor sites, primarily the hard palate and tuberosity, for mucogingival surgeries around natural teeth.

## 2. Materials and Methods

This systematic review was prepared in accordance with the PRISMA (Preferred Reporting Items for Systematic Reviews and Meta-Analyses) statement based on the proposed focused question [13], the PRISMA 2020 checklist is available as a Supplementary Materials Table S1. This review was retrospectively registered in PROSPERO under the ID CRD420251030155 after the initiation of screening in early 2025. The final database search was completed on 31 December 2023, and initial title and abstract screening began in January 2024. Although this registration occurred after the identification of eligible studies had begun, all inclusion criteria, outcomes of interest, and synthesis plans were established prior to screening and strictly followed throughout the review process. No deviations from the original protocol occurred.

### 2.1. PICO Question

(Population, Intervention, Comparison, Outcome)

P: Harvested soft tissue grafts in humans

I: Hard palate

C: tuberosity

O: Graft dimension, tissue thickness, post-operative pain/discomfort

Primary focus question: “In medically fit individuals with indication for intra-oral soft tissue grafting, which intraoral donor site will provide superior clinical outcomes and less patient discomfort?”

### 2.2. Main Outcomes

The primary outcomes of this systematic review focused on three key aspects of soft tissue grafting: graft dimension, tissue thickness, postoperative pain and discomfort levels. Graft dimension availability was assessed by measuring the thickness and size of harvested grafts. Tissue thickness was evaluated by comparing initial graft dimensions to those measured after healing, assessing the stability of the graft over time. Finally, postoperative pain and discomfort were evaluated using patient-reported outcomes.

### 2.3. Eligibility Criteria

The inclusion criteria were clinical studies and clinical trials with medically fit adult subjects, studies that are published in English language, studies that report any of our predetermined outcomes and exclusively performed around teeth. The exclusion criteria included editorials, reviews, case reports, case series, experimental, and in vitro studies. Studies conducted on medically compromised patients, soft tissue grafting procedures around dental implants or for ridge augmentation, and those not reporting relevant outcome data were also excluded.

### 2.4. Search Strategy

A systematic electronic search was conducted in PubMed, Web of Science, OVID Medline, and Scopus, by applying a grouping of keywords and MeSH terms. The search terms are provided in Table 1. In this study, only human studies, published in dental journals and in English from inception through December 2023 were examined and the final search was conducted on 31 December 2023. The screening process consisted of three stages that were conducted by two independent researchers. In case of disagreement regarding the inclusion or exclusion criteria, a discussion was carried out to resolve the differences. In the event of a lack of consensus, the decision was made by a third party (a senior researcher).

### 2.5. Paper Selection

In the first stage, the two researchers independently reviewed all titles from the electronic search to determine whether the papers were relevant or not. During the second stage, the abstracts of the pre-identified papers were screened by the researchers independently in order to exclude those articles that did not meet the inclusion criteria. The third stage involved a full-text reading of the selected articles to verify their outcome eligibility.

### 2.6. Data Collection

Qualitative and quantitative data were collected from the studies, including: (1) general study characteristics and basic demographic data of subjects (author, year of publication, number of groups studied, sample size). (2) surgical procedure and donor site (recipient site preparation, donor site, graft dimension). (3) outcome variables of interest (graft dimension availability, tissue thickness, percentage of root coverage, data on keratinized tissue changes, and post-operative pain and discomfort level).

### 2.7. Risk of Bias (Quality) Assessment

Investigators independently assessed the risk of bias using the Revised Cochrane Risk of Bias tools (RoB-2 for randomized controlled trials, and ROBINS-I for non-randomized studies) and resolved conflicts through discussion. The Bias domains for ROB 2 were due to: randomization process (D1), deviations from intended interventions (D2), missing outcome data (D3), outcome measurement (D4), and selection of the reported result (D5). The study was considered to be at “low risk of bias” if all domains were low, “raised some concerns/moderate risk” if at least one domain raised some concern for this result, and at “high risk” If there was a high risk of bias in at least one domain or had some concerns for multiple domains. On the other hand, ROBINS-I evaluates potential bias through seven domains, including: confounding, selection of participants, intervention classification, deviation from intended intervention, missing data, measurement of outcomes, and selection of the reported result. Cochrane Collaboration tool (robvis) (version 2) was used to generate the quality risk-of-bias assessment results’ chart [14].

### 2.8. Statistical and Data Analysis

The findings of each study were combined in a descriptive style, emphasizing the main outcomes for each donor site. This approach provided a clear comparison of graft dimensions, tissue thickness, and postoperative pain levels between the palate and tuberosity, enabling a comprehensive understanding of their clinical performance.

A meta-analysis was considered during protocol development. However, due to substantial heterogeneity in study design, outcome measures, follow-up duration, and methodological quality, a quantitative synthesis was not feasible. Specific outcomes such as changes in gingival thickness (GT) and patient-reported pain were evaluated for potential pooling. However, differences in outcome definitions, measurement tools, timepoints, and reporting formats precluded meaningful aggregation. Therefore, a narrative synthesis was conducted for all included studies.

We mapped feasibility by outcome and timepoint, considering variation in measurement tools, follow-up durations, and outcome definitions. A detailed justification per outcome is presented in Section 3.

## 3. Results

### 3.1. Study Selection

**Figure 1:**
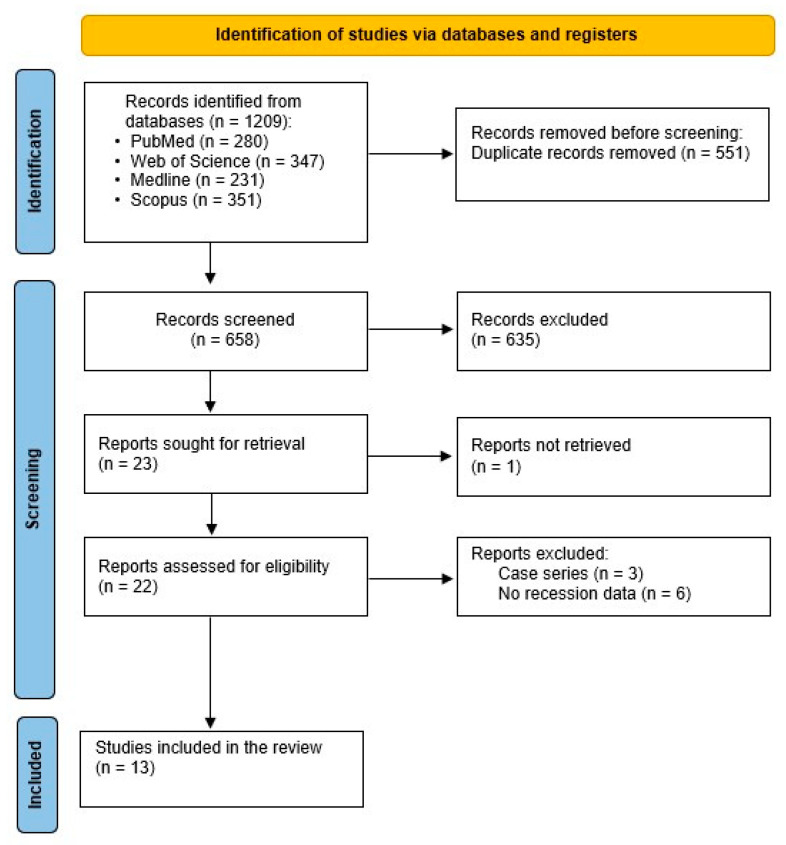
Flow chart showing the article selection process PRISMA.

The preliminary search produced a total of 1209 records, comprising 280 papers from PubMed, 347 from Web of Science, 351 from Scopus, and 231 from Medline. Out of these, 551 papers were initially removed due to duplication. Subsequently, 658 records were evaluated based on their titles and abstracts, resulting in the elimination of 635 records. After excluding studies that did not meet the inclusion criteria, 23 papers were sought for retrieval, with one article that could not be retrieved. As a result, 22 full texts were assessed for eligibility. The final reviewed studies included in the present systematic review were 13 papers: seven were randomized clinical trials, one clinical study, three prospective clinical studies, one observational clinical study, and one split-mouth clinical study. The workflow for the systematic review is shown in Figure 1. The final included studies with their characteristics and outcomes are summarized in Table 2 and Table 3.

### 3.2. Soft Tissue Grafts Harvested from the Tuberosity Area

#### 3.2.1. Graft Dimension

Only one study, by Amine et al., utilized both the palate and the tuberosity for harvesting tissue grafts. All grafts in the study were harvested with a standardized thickness of 1.5 mm for both free gingival graft (FGG) and connective tissue graft (CTG) with coronally advanced flap (CAF) groups. For the CAF and CTG cases, a thickness of 1.0 mm was maintained after de-epithelialization [2].

#### 3.2.2. Tissue Thickness

In the same study by Amin et al., shrinkage in the buccolingual dimension was detected and expressed as thickness. Palatal donor sites may experience more volume or dimensional changes due to adipose tissue in the submucosa. The thickness of healed grafts at 8 weeks post-operatively was greater in tuberosity in both FGG and CTG groups. The mean gingival thickness of the healed tuberosity grafts in FGG and CTG groups were 2.9 ± 0.5 mm and 2.7 ± 0.7 mm, respectively, versus healed palatal grafts thickness 2.3 ± 0.6 mm and 2.1 ± 0.7 mm, respectively, indicating superiority in favor of tuberosity grafts. In other words, tissue stability or volume reduction varied based on the donor location rather than initial thickness.

No substantial difference was observed in the mean percentage of root coverage (RC) from tuberosity or palatal donor sites (67 ± 12% against 62 ± 13%, respectively, *p* = 0.102), and the final thickness of healed graft has no significant role in the percentage of the RC. Notably, the short-term follow-up period of 8 weeks and the reliance on subjective pain reporting represent key limitations of this study [2].

#### 3.2.3. Post-Operative Pain/Discomfort

In terms of postoperative pain, it was significantly lower at the tuberosity donor site compared to the palatal donor site during the first two weeks. Patients reported a mean pain level of 2.6 at tuberosity sites versus 5.9 at palatal sites using a 10-point scale [2].

### 3.3. Soft Tissue Grafts Harvested from the Hard Palate

#### 3.3.1. Graft Dimension

Four studies did not mention any dimensions related to the graft after harvesting [15,16,20,24]. Most papers mentioned that 1–1.5 mm was the average of their graft thickness [2,17,18,19,21,22,25,26]. However, Lafzi et al. used a CTG with a thickness of less than 1 mm (0.9 mm) [23].

#### 3.3.2. Tissue Thickness

Some studies documented the changes in gingival thickness (GT) after utilizing autogenous soft tissue grafts of different approaches. Di Domenico et al. reported a change in marginal tissue thickness (MTT) of 1.47 ± 0.77 mm [15], while Elmahdi et al. noted a change in GT of 0.94 ± 0.52 mm at nine months post-operatively [19]. GT increase ranged from 0.5 to 1.11 mm [16,19,22], and could reach up to 2.3 mm in one study [2]. In addition, the gingival/keratinized tissue width (KTW) at different follow-up periods was reported among the included studies ranging from 1.15 up to 5.2 mm [2,15,17,18,19,20,22,23,25,26].

Measurements of recession at both baseline and follow-up were documented, except for one study by Amin et al. that did not report baseline recession data [2]. Mean percentage of root coverage (RC%) ranged from 62 to 98.9% [2,15,17,19,22,23,24,25,26], and average root coverage (ARC%) ranged from 89.90 to 95.77% [18,20].

#### 3.3.3. Post-Operative Pain/Discomfort

Four studies did not record patient-reported outcomes [15,18,20,26]. While another four studies used the Visual Analog Scale (VAS) to evaluate post-operative pain [17,19,21,22], and the remaining three studies used questionnaires to rate the discomfort levels [2,23,24,25]. No significant difference was found in the pain/discomfort level among different harvesting techniques used at the palatal donor sites [19,21]. Except in one study, Harris RJ, which compared free gingival graft knife harvesting method (FGGK) with the parallel incisions method (PI). The study observed that PI produced less postoperative discomfort compared to the FGGK [25].

Comparing pain/discomfort level of autogenous grafts versus soft tissue substitutes; Aroca et al. observed that patient complaint was higher, and satisfaction was lower in the CTG group as opposed to the Collagen matrix (CM) group (12.8 ± 7.5, 90.6 ± 7.9 vs. 7.3 ± 3.4 and 92.9 ± 8.4, respectively) at 12 months. Although the satisfaction percentage was 100% in the CM group, the difference was not statistically significant [22].

In the study by Elmahdi et al. [17] which compared two groups, connective tissue graft (CTG) and acellular dermal matrix (ADM), the CTG group experienced significantly higher pain levels during the first four postoperative days. However, there was no significant difference in patient satisfaction between the two groups at the 9-month follow-up (ADM: 8.24 ± 0.43, CTG: 8.24 ± 0.65)

### 3.4. Risk of Bias Assessment

#### 3.4.1. ROB 2 (Randomized Trials)

The ROB 2 assessment for the randomized trials included in this review revealed generally favorable methodological quality. Most studies exhibited low risk of bias across all five ROB 2 domains. However, Domain 1 (bias arising from the randomization process) and Domain 4 (bias in measurement of the outcome) showed “some concerns” in two studies [2,20], primarily due to limited reporting on allocation concealment and blinding. The remaining domains (D2, D3, and D5) were rated as low risk in all five studies. Studies by Wilson et al. [24], Elmahdi et al. [17], Bakhishov et al. [19], Pandit et al. [21], Aroca et al. [22], and Lafzi et al. [23] achieved overall low risk of bias ratings.

#### 3.4.2. ROBINS-I (Non-Randomized Studies)

The ROBINS-I assessment indicated that most non-randomized studies had a low overall risk of bias. However, moderate risk was identified in Domain 1 (bias due to confounding) in two studies [16,25]. Additional moderate risk ratings were noted in Domain 2 (selection of participants) and Domain 4 (deviations from intended interventions) in one study [16], and in Domains 6 and 7 (measurement of outcomes and selection of reported result) in another [25]. The remaining domains in these studies were judged as low risk. Studies by Di Domenico et al. [15], Bednarz et al. [18], and Wennstrom and Zucchelli [26] were judged to have low risk of bias across all domains. In contrast, studies by Farkhshatova et al. [16] and Harris [25] presented moderate overall risk due to the absence of a control group, potential for confounding, and limited reporting on blinding.

**Figure 2:**
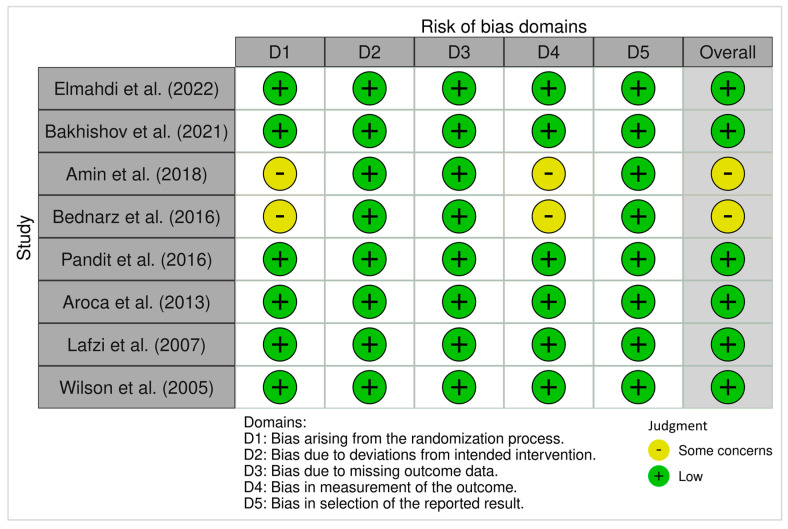
ROB 2 Traffic-light plots of risk-of-bias in randomized trials using the Cochrane Risk of Bias Tool. Risk of Bias Summary presents the review authors’ judgments regarding each domain of bias across included studies, allowing for a direct visual comparison between studies and bias categories. Each cell in the matrix is marked with a green, yellow, or red symbol, indicating low, moderate, or high risk of bias, respectively [2,17,19,20,21,22,23,24].

**Figure 3:**
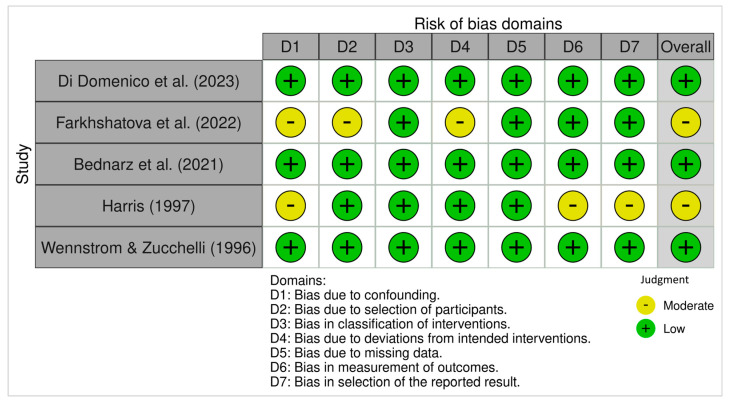
ROBINS-I Traffic-light plots of risk-of-bias judgments for non-randomized studies of interventions using the Cochrane Risk of Bias Tool. Each cell in the matrix is marked with either a green, yellow, red, or dark symbol, indicating low, moderate, serious, or critical risk of bias, respectively [15,16,18,25,26].

Figure 2 and Figure 3 display a summary of risk of bias, which presents the review authors’ judgments regarding each domain of bias across all included studies, allowing for a direct visual comparison between studies and bias categories. Each cell in the matrix is marked with a green, yellow, or red symbol, indicating low, moderate, or high risk of bias, respectively.

**Figure 4:**
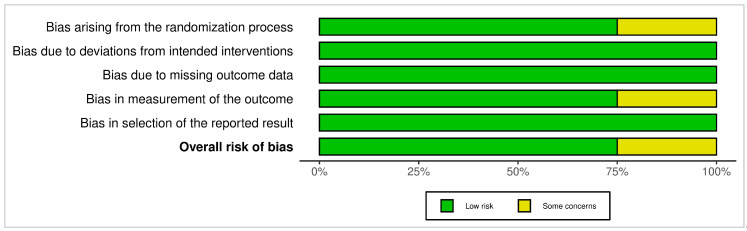
ROB 2 Bar graph: the distribution of risk of bias across key methodological domains assessed using the Cochrane Risk of Bias Tool for randomized clinical studies.

**Figure 5:**
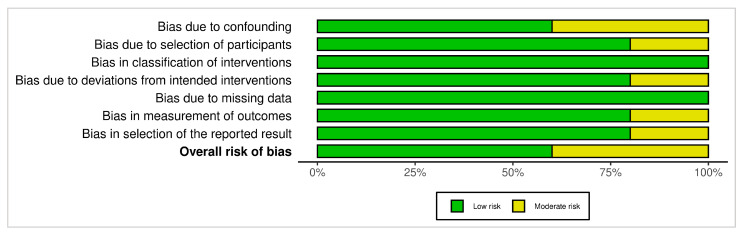
ROBINS-I Bar graph: the distribution of risk of bias across key methodological domains assessed using the Cochrane Risk of Bias Tool for non-randomized studies of interventions.

Figure 4 and Figure 5 present bar graphs showing the distribution of risk of bias across key methodological domains assessed using the Cochrane Risk of Bias Tools (ROB 2 and ROBINS-I).

Due to heterogeneity in outcome measures, timepoints, and reporting formats, no meta-analysis was performed. VAS pain scores were reported using different scales and at varying postoperative timepoints, while gingival thickness outcomes varied in measurement methods and anatomical landmarks. Only one study directly compared tuberosity vs. palate donor sites, preventing meaningful comparative synthesis. Consequently, a narrative synthesis was considered the most appropriate approach.

### 3.5. The Heterogeneity Across Studies by Each Major Outcome and Timepoint

1. Gingival Thickness (GT)

Measurement Tools: Inconsistent methods were used to assess GT (e.g., periodontal probes, ultrasound, or calipers). Some studies reported mean GT in mm, others used histologic measures or provided ΔGT.

Timepoints: Timepoints for follow-up varied widely ranging from 6 weeks (Amin et al.) [2] to 9 months (Elmahdi et al.) [17] to 12 months and beyond in others.

Heterogeneity Summary: The variability in follow-up duration and lack of standardized measurement tools precluded pooling GT data meaningfully.

2. Keratinized Tissue Width (KTW)

Measurement Tools: KTW was generally reported in mm, but baseline and follow-up durations were inconsistent.

Timepoints: KTW was reported at 3, 6, 9, 12, and even 36 months across studies. No subset of studies had a consistent follow-up window to enable aggregation.

Heterogeneity Summary: Due to inconsistency in follow-up timeframes and reporting formats (some reported mean differences, others only post-treatment values), meta-analysis was not feasible.

3. Patient-Reported Outcomes (Pain/Satisfaction—VAS or Questionnaire)

Measurement Tools: Some studies used a VAS (0–10), others used a 4-point scale, verbal rating scales, or unvalidated questionnaires.

Timepoints: Pain was recorded at varied intervals, some daily for the first 14 days (e.g., Elmahdi et al.) [17], others at 1, 2, or 8 weeks.

Heterogeneity Summary: The heterogeneity in pain assessment tools and the timing of evaluations made it impossible to statistically combine the data.

4. Clinical Outcomes (Recession Coverage, %RC, %CRC)

Measurement Tools: Although %RC and %CRC were frequently reported, some studies only reported raw recession depth or width, and the reference points for these calculations varied.

Timepoints: Follow-ups ranged from 6 weeks to 3 years. No uniform timepoint was reported across more than 2–3 studies.

Heterogeneity Summary: The clinical endpoint inconsistencies (RC vs. CRC vs. MRC), measurement variability, and variable follow-up durations limited the ability to pool results reliably.

## 4. Discussion

The present systematic review aimed to evaluate the clinical and patient-reported outcomes of mucogingival defects treated with intraoral soft tissue grafts, mainly those harvested from the hard palate and tuberosity area. The findings of this review showed that all the studies used palatal grafts either as controls for soft tissue substitutes, or for comparing different harvesting techniques. The exception was the study conducted by Amin et al.; it was the only clinical study comparing the palatal versus tuberosity grafts [2]. In addition, the post-operative follow-up periods varied among the selected studies, and clinical outcomes were recorded at different intervals, ranging from 14 days up to 3 years.

Our findings indicate that tuberosity donor sites led to significantly lower postoperative pain during the initial two postoperative weeks compared to palatal donor sites (2.6 ± 2.16 versus 5.9 ± 2.74, *p* < 0.001). Yet both donor sites were healed with secondary intention, pain remained less with tuberosity grafts, and this could be attributed to the fact that the connective tissue left over the bone is thicker than that at the palatal donors [27]. Also, the location of tuberosity away from the friction with food or tongue during function serves as another reason for that reduced pain [2]. This aligns with Tavelli et al.’s conclusions about minimized morbidity of tuberosity grafts, attributable to decreased intra- and postoperative complication rates in returns contributing to patient satisfaction [3].

Although donor site tuberosity was associated with less pain compared to palatal grafts, numerous ways for obtaining CTGs have evolved, intending to improve clinical outcomes. The harvesting technique of subepithelial connective tissue graft (SCTG) plays a role in minimizing post-surgical pain, and some harvesting methods showed superiority compared to other techniques across various dimensions. Harris found that the parallel incisions (PI) method resulted in reduced patient discomfort, smaller wounds at one week postoperatively, a more uniform graft, and greater ease of clinical application in comparison to the free gingival graft knife method (FGGK). However, no statistically significant difference in mean root coverage was obtained from various graft harvesting techniques (98.3% to 98.7%) [25].

Langer and Langer’s approach showed superiority over the Unigraft knife technique for procuring the connective tissue graft. The wound in the Langer and Langer trap door procedure healed marginally faster than the Unigraft knife method, potentially resulting in less patient discomfort. Conversely, the Unigraft knife approach yielded a slightly bigger wound area at the palate. Additionally, it is technique-sensitive and poses challenges when applied in high and small palatal vaults. However, both techniques proved effective in achieving root coverage outcomes [21].

In the current review, the reported thickness of healed grafts at 8 weeks postoperatively was greater for the tuberosity in both FGG and CTG groups. The mean gingival thickness of healed tuberosity grafts was 2.9 ± 0.5 mm (FGG) and 2.7 ± 0.7 mm (CTG), compared to 2.3 ± 0.6 mm and 2.1 ± 0.7 mm, respectively, for palatal grafts. While this may indicate a trend toward greater graft thickness with tuberosity donor sites, these findings are limited to a single clinical study and should be interpreted with caution.

To further contextualize this observation, Dellavia et al. [28] reported volumetric behavior of connective tissue grafts from the palate and tuberosity in a ridge augmentation setting. Although this study falls outside our eligibility criteria and was not included in the evidence synthesis, it offers histological perceptions about tissue stability and healing characteristics. Specifically, tuberosity grafts demonstrated greater tissue volume and a tendency toward hyperplastic response compared to palatal grafts, which showed some volume reduction but no hyperplasia. These findings may reflect intrinsic histological differences such as lamina propria composition, collagen cross-linking, and fibroblast maturation that influence graft behavior [10,11,12,28].

Bakhishov et al. assessed the histological differences in the palatal grafts harvested either as de-epithelialized gingival graft (DGG) or subepithelial connective tissue graft (SCTG) using single incision (SI) method, accompanied by tunneling technique (TUN) to treat multiple adjacent gingival recessions. The study reported that DGG specimens exhibited partial epithelial remnants within the superficial strata. In regard to cellularity, SCTG demonstrated a higher degree of cellularity relative to the DGG. Concerning vascularization, no statistically significant difference was identified among the groups [19].

Azar et al. conducted a histological and histomorphometric comparison of subepithelial connective tissue grafts (SCTGs) obtained from palatal mucosa using two harvesting techniques: mucoperiosteal-total thickness (including lamina propria and complete submucosa with periosteum) and mucosal-partial thickness (comprising lamina propria and a portion of the submucosa). The ratios of adipose tissue (AT), connective tissue proper (CTP), and vascular tissue (VT) were assessed. It was found that SCTGs collected by the mucosal technique possess a higher proportion of connective tissue proper (CTP) and a lower proportion of adipose tissue (AT), compared to those acquired via the mucoperiosteal technique, while vascular tissue (VS) remained consistent across both methods [29].

Various alternatives to autogenous grafts have emerged for the sake of improving patients’ experience and clinical outcomes, such as acellular dermal matrix (ADM), collagen matrix (CM), and Fascia Lata (FL) allografts. In Aroca et al.’s study comparing CM with CTG in the management of Miller Class I and II when utilizing modified coronally advanced tunnel method (MCAT), the data suggested that the application of collagen matrix (CM) may serve as an alternative to CTG by decreasing surgical duration and patient morbidity; nevertheless, it resulted in lower CRC compared to CTG (42% and 85%, respectively, *p* < 0.05) [22].

Another study evaluated two grafting materials: SCTG and ADM alongside MCAT for the treatment of multiple adjacent recessions; the control cohort (CTG) exhibited a greater enhancement in KTW (1.15 ± 1.16 mm vs. 0.21 ± 0.84 mm) and a notable increase in GT (0.94 ± 0.52 mm vs. 0.53 ± 0.41 mm). Nevertheless, the variations in gingival recession width reduction, probing depth alterations, and clinical attachment loss improvement did not reveal significant differences among the respective groups. In terms of postoperative pain level, it was higher with CTG in the first four days, and patient satisfaction with esthetic outcomes nine months post-treatment was comparable between the groups (VAS score: 8.24 ± 0.43 for ADM, and 8.24 ± 0.65 for CTG) (*p* = 0.99) [17].

A previous Spanish systematic review [30] assessed the clinical outcomes of periodontal and peri-implant plastic surgery with autogenous graft obtained from the palate versus the tuberosity. However, their review included a limited number of studies and lacked patient-reported outcomes The review concluded that both donor sites yield comparable clinical results with no statistically significant differences. Yet, tuberosity graft notably enhances the gingival phenotype of the recipient region, with lower postoperative pain during the initial weeks of healing.

Konflanz et al. 2021, in turn, presented a systematic review and meta-analysis restricted to randomized controlled trials of single recession defects published up to 2019, and they explicitly highlighted the absence of RCTs directly comparing tuberosity and palatal donor sites [31]. In contrast, our review adopted a broader scope by including both randomized and non-randomized studies, evaluating not only clinical outcomes but also patient-reported outcomes (PROs), and applying the updated PRISMA 2020 methodology. These differences allow our work to complement and expand upon earlier reviews, while also emphasizing the persistent knowledge gap regarding direct comparative evidence for tuberosity grafts.

The methodological quality of the included studies, as assessed using the ROB-2 and ROBINS-I tools, was generally favorable. Most randomized trials demonstrated low risk of bias across domains, with only two studies [2,20] showing concerns related to outcome assessment blinding and allocation concealment. Similarly, most non-randomized studies achieved low risk ratings, except for moderate concerns in confounding and participant selection in two studies [16,25]. These findings enhance the credibility of the included evidence, although some caution is required when interpreting results from observational data due to their inherent limitations.

In this review, a descriptive (narrative) synthesis was conducted instead of a meta-analysis due to significant clinical and methodological heterogeneity among the included studies.

The variability in graft harvesting techniques, surgical protocols, outcome measures, and follow-up durations made it challenging to combine their findings into a single pooled estimate without risking misleading conclusions. Additionally, inconsistent reporting of data, such as differences in pain measurement tools (e.g., VAS types and timing), methods of assessing graft thickness, and considerable variation in follow-up periods (ranging from 2 weeks to several years), further prevented the reliable pooling of data for meta-analysis. Also the risk of bias assessments revealed significant methodological differences between the studies such as the lack of randomization in some studies or inconsistent blinding methods which further complicate the aggregation of results, as the validity of pooled estimates would be questionable without addressing these biases; the variations in the follow-up periods across studies (2 weeks or years), adds another layer of complexity to meta-analysis, as it introduces considerable disparity in the time frame over which outcomes were measured.

Although meta-analysis is often valuable in strengthening evidence synthesis, in this case, it was not feasible. Only one study [2] directly compared tuberosity and palatal donor sites, and key outcomes could not be reliably aggregated. This highlights the need for more standardized outcome reporting and high-quality, controlled comparative trials to enable meaningful quantitative synthesis in the future.

The limitation of this systematic review is that it was registered retrospectively in PROSPERO after the initiation of screening and the limited availability of high-quality comparative studies, with only one split-mouth clinical trial exclusively focused on soft tissue grafting around natural teeth. Additionally, several included studies lacked detailed reporting on key clinical variables, such as recession characteristics, graft dimensions, and patient-reported outcomes, restricting the depth and consistency of the analysis. Although all eligible studies were included in this review based on the predefined inclusion criteria, some had relatively short follow-up durations (≤2 months). While these were analyzed for completeness, their findings should be interpreted with caution, as they may not reliably reflect the long-term stability of outcomes. Future studies with extended follow-up are therefore recommended to provide stronger evidence.

Only one included clinical study (Amin et al. [2]) directly compared tuberosity and palatal donor sites. While this study reported significantly lower postoperative pain and favorable healing outcomes for the tuberosity group, its short follow-up period and single-center design limit the generalizability of these findings. As such, conclusions regarding the superiority of tuberosity donor sites remain preliminary and should be interpreted with caution.

To strengthen the existing evidence base, future research should prioritize well-designed randomized controlled trials with standardized methodologies, comprehensive outcome reporting, and extended follow-up periods. Patient-centered metrics, including discomfort, esthetic satisfaction, color matching and functional outcomes, should also be emphasized to support evidence-based and individualized donor site selection in clinical practice.

## 5. Conclusions

Within the given limitations, it can be concluded that the quality of the harvested graft plays a critical role in determining successful clinical outcomes, such as root coverage and increased keratinized tissue width and thickness. The hard palate remains a well-established and reliable source of soft tissue grafts, offering consistent graft dimensions and favorable healing characteristics. Since the direct comparative evidence between tuberosity and palatal donor sites is derived from a single short-term study, the conclusions regarding the improved patient-reported outcomes following tuberosity grafts need to be interpreted with caution and be further investigated. As such, no definitive conclusions can be drawn regarding the superiority of one donor site over the other. This review instead highlights a significant gap in the literature and recommends well-designed, high-quality randomized controlled trials with long-term follow-up to enable evidence-based selection of optimal donor sites in periodontal plastic surgery.

## Figures and Tables

**Table 1:** Search terms used among different databases.

Database	Keywords
PubMed	((“soft tissue” OR “free gingival grafting” OR “gingival grafting” OR “connective tissue graft”) AND (“Tissue Donors” OR Tuberosity OR Palate) AND graft)
Web of Science	(TS = (“soft tissue” OR “free gingival grafting” OR “gingival grafting” OR “connective tissue graft”)) AND (TS = (“Tissue Donors” OR Tuberosity OR Palate)) AND TS = (Graft)
OVID Medline	(TS = (“soft tissue” OR “free gingival grafting” OR “gingival grafting” OR “connective tissue graft”)) AND (TS = (“Tissue Donors” OR Tuberosity OR Palate)) AND TS = (Graft)
Scopus	(TITLE-ABS-KEY (“soft tissue” OR “free gingival grafting” OR “gingival grafting” OR “connective tissue graft”)) AND (TITLE-ABS-KEY (“Tissue Donors” OR tuberosity OR palate)) AND (TITLE-ABS-KEY (Graft))

**Table 2:** Study characteristics of included papers.

Title	Author	Year	Study Type	Population Sample	Control Group	Test Group	Miller Class
Multiple Coronally Advanced Flap with a Selective Use of Connective Tissue Graft: A 3-Year Prospective Clinical and Histological Study	Giovanna Laura Di Domenico et al. [15]	2023	non-randomized prospective clinical trial + histological study	23 patients, total of 93 recession (59 in maxilla, 34 mandible)	MCAF alone (39 sites)	MCAF + CTG (54 sites)	RT 1
Diagnosis and Treatment of Miller Class I Gingival Recession Using Free Connective Tissue Autograft from the Hard Palate. Clinical Observation.	Rushana Farkhshatova et al. [16]	2022	observational clinical study	33 patients	No	patients with Miller class I, maximum no. of recessions 6 teeth per patient and minimum 1 tooth.	Class I = 24 recession zones
Evaluation of Subepithelial Connective Tissue Graft Versus Acellular Dermal Matrix with Modified Coronally Advanced Tunnel Technique in Treatment of Multiple Gingival Recessions: A Randomized, Parallel Design Clinical Trial	Fatema Elmahdi et al. [17]	2022	parallel group randomized controlled clinical trial	24 patients	MCAT-SCTG modified coronally advanced tunnel with subepithelial connective tissue graft	MCAT-ADM acellular dermal matrix	RT1—facial maxillary or mandibular multiple adjacent gingival recessions
Coronally Advanced Flap in the Treatment of Multiple Adjacent Gingival Recessions Along with a Connective Tissue Graft Harvested from Augmented or Nonaugmented Palatal Mucous Membrane: A Two-Year Comparative Clinical Evaluation	Wojciech Bednarz et al. [18]	2021	prospective non- randomized comparative clinical trial	35 patients with a total of 148 MAGRs	(NA-CTG + CAF) 71 gingival recessions treated with the nonaugmented connective tissue graft	(A-CTG + CAF) 77 recessions treated with augmented CTG with coronally advanced flap	Class I. 70 sites II. 60 III. 18
De-Epithelialed Gingival Graft Versus Subepithelial Connective Tissue Graft in the Treatment of Multiple Adjacent Gingival Recessions Using the Tunnel Technique: 1-Year Results Of A Randomized Clinical Trial	Hikmat Bakhishov et al. [19]	2021	single-blinded, randomized clinical trial	27 patients	SCTG + TUN subepithelial connective tissue graft with tunnel technique	DGG + TUN de-epithelialized gingival graft with the tunnel technique	class I and/or II (RT1)
Tuberosity Versus Palatal Donor Sites for Soft Tissue Grafting: A Split-Mouth Clinical Study	Peter Amin et al. [2]	2018	split-mouth, randomized clinical study	20 participants, 10 for FGG, and 10 for CTG + CAF. Bilateral soft tissue grafting from tuberosity and palate carried in each patient, and served as their control	Tuberosity grafts (total 20 site): 10 FGG, 10 CTG	Palatal grafts (total 20 sites): 10 FGG, 10 CTG	Class I, II, or III, minimum of 2 mm recession
A Preliminary Clinical Comparison of the Use of Fascia Lata Allograft and Autogenous Connective Tissue Graft in Multiple Gingival Recession Coverage Based on the Tunnel Technique	Wojciech Bednarz et al. [20]	2016	comparative randomized clinical trial	30 patients	(MCAT/CTG) modified coronally advanced tunnel technique with connective tissue graft (*n* = 40)	(MCAT/FL) Fascia Lata Allografts using a modified coronally advanced tunnel technique (*n* = 97)	Class I = 125 Class II = 12
Comparison of Two Techniques of Harvesting Connective Tissue and its Effects on Healing Pattern at Palate and Recession Coverage at Recipient Site	Nymphea Pandit et al. [21]	2016	randomized clinical study	16 patients with 30 sites	Group I: CTG harvested using Unigraft Knife	Group II, CTG harvested by the Langer and Langer technique	Class I and II (recession ≥ 2 mm)
Treatment of Multiple Adjacent Miller Class I And II Gingival Recessions with a Modified Coronally Advanced Tunnel (MCAT) Technique and a Collagen Matrix or Palatal Connective Tissue Graft: A Randomized, Controlled Clinical Trial	Salvi Arocav et al. [22]	2013	randomized, controlled, split-mouth clinical trial	22 patients, 156 recessions	78 sites Modified Coronally Advanced Tunnel (MCAT) + (CTG)	78 sites MCAT + bioresorbable collagen matrix (CM)	Class I and II
Effect of Connective Tissue Graft Orientation on the Root Coverage Outcomes of Coronally Advanced Flap	Ardeshir Lafzi et al. [23]	2007	randomized controlled clinical trial	8 patients with a total of 16 bilateral recessions	P-teeth: periosteum contacting the tooth surface	P-flap: periosteum contacting the flap	Class I and II
Evaluation of the Safety and Efficacy of Periodontal Applications of a Living Tissue-Engineered Human Fibroblast derived Dermal Substitute. II. Comparison to the Subepithelial Connective Tissue Graft: A Randomized Controlled Feasibility Study	Thomas Wilson et al. [24]	2005	randomized controlled split-mouth study	13 patients	CAF + SCTG	CAF + human fibroblast-derived dermal substitute (HF-DDS) either a single or double layer (8 sites, 5 sites, respectively)	Class I or II
A Comparison of Two Techniques for Obtaining a Connective Tissue Graft from the Palate	Randali J. Harris [25]	1997	non-randomized clinical trial	26 patients	13 patients with 19 defects treated with free gingival graft knife harvesting method (FGGK)	13 patients with 15 defects treated with parallel incisions harvesting method (PI)	Class I or II
Increased Gingival Dimensions. A Significant Factor for Successful Outcome of Root Coverage Procedures? A 2-Year Prospective Clinical Study	Jan L. Wennstrom and Giovanni Zucchelli [26]	1996	non-randomized prospective clinical study	67 patients, 103 recessions	CAF alone (*n* = 45)	CAF + CTG (*n* = 58)	Class I

Abbreviations: MCAF: Multiple Coronally Advanced Flap, CTG: connective tissue graft, CAF: coronally advanced flap, SCTG: Subepithelial Connective Tissue Graft, ADM: Acellular Dermal Matrix, MCAT: Modified Coronally Advanced Tunnel, CM: collagen matrix, MCAT/FL: Fascia Lata Allografts using a modified coronally advanced tunnel, NA-CTG: non augmented Connective Tissue Graft, A-CTG: augmented Connective Tissue Graft, FGGK: free gingival graft knife harvesting, HF-DDS: human fibroblast-derived dermal substitute.

**Table 3:** Summary of Studies’ Outcomes.

Reference	Recipient Site	Donor Site	Graft Dimension	Rec. Dimension at Baseline	Rec Dimension at F.U	Other Findings	Patient Reported Outcome/Satisfaction	Follow Up Period
Di Domenico et al. (2023) [15]	CAF (split-full-split) with/out CTG	palate—no details	not mentioned	mean REC: -MCAF 1.97 ± 0.87 -MCAF + CTG 2.91 ± 1.01 (significantly lower at MCAF)	MCAF RecRed 1.69 ± 0.81 %RC 93 ± 21 CRC 1 yr 90% CRC 3 yr 86% MCAF + CTG RecRed 2.69 ± 1.08 %RC 93 ± 16 CRC 1 yr 90% CRC 3 yr 81%	mean MCAF KTW0 2.39 ± 1.02 KTW 3 yr 3.19 ± 1.21 ΔKTW 0.72 ± 1.11 ΔMTT 0.22 ± 0.89 mean MCAF + CTG KTW0 1.74 ± 0.89 KTW 3 yr 3.33 ± 1.2 ΔKTW 1.69 ± 1.59 ΔMTT 1.47 ± 0.77	not mentioned	1 and 3 years
Farkhshatova et al. (2022) [16]	Tunnel—two-layer technique	palate —CTG harvested according to the standard technique, one case as example was DGG	not mentioned	Rec height: 4.0 ± 0.2 mm	Rec height: at 14 days 0.8 ± 0.07 at 1 month 1.1 ± 0.04 In 3 patients, the height of residual recession 1 month after treatment was 1.5 ± 0.03 mm in the lower ant. area	Thickness of KG: At baseline 0.79 ± 0.09 at 14 days 2.8 ± 0.03 at 1 month 1.9 ± 0.08 KG increase: at 14 days 3.2 ± 0.13	4-point scale (0–3). Highest pain levels on the first day after surgery (−1.78 ± 0.29 points), decreasing significantly by days 3–7 (0.2 ± 0.11 points), and completely absent by days 7–14.	day 7, 14, then after 1 and 3 months
Elmahdi et al. (2022) [17]	modified coronally advanced tunnel (MCAT) technique + CTG	Palate, single incision method	-CTG thickness 1 to 1.5 mm -ADM height of 6 to 7 mm apico-coronal from CEJ	ADM GRD0 2.87 ± 0.31 GRW0 3.28 ± 1.44 CTG GRD0 2.76 ± 0.89 GRW0 4 ± 1.48	9 M ADM RD 0.76 ± 0.1 RW 0.87 ± 0.70 RD reduction 2.10 ± 0.64 RW reduction 2.41 ± 1.19 RC% 72.72 ± 23.36 9 M CTG RD 0.53 ± 0.48 RW 1.59 ± 1.96 RD reduction 2.23 ± 0.68 RW reduction 2.41 ± 1.94 RC% 82.62 ± 16.30	ADM KTW0 3.03 ± 0.72 KTW9 3.12 ± 0.69 KTWΔ 0.21 ± 0.84 GT0 1.10 ± 0.20 GT9 1.65 ± 0.39 GTΔ 0.53 ± 0.41 CTG KTW0 2.65 ± 0.92 KTW9 3.82 ± 1.3 KTWΔ 1.15 ± 1.16 GT0 1.33 ± 0.54 GT9 2.26 ± 0.63 GTΔ 0.94 ± 0.52	Patient esthetic satisfaction and postoperative pain recorded using VAS. CTG group significantly higher pain levels during the first four postsurgical days. Patient satisfaction at 9 months: no difference btw grp (ADM 8.24 ± 0.43, CTG 8.24 ± 0.65)	daily till day 14, then at 9 months
Bednarz et al. (2021) [18]	CAF (trapizoidal flap) + CTG	palate—trap door	about 1 mm in thickness	Control (NA-CTG + CAF) GRD 3.08 ± 1.14 GRW 3.64 ± 1.25 Test (A-CTG + CAF) GRD 2.72 ± 1.00 GRW 3.47 ± 0.92	Control (NA-CTG + CAF)) 6 M GRD 0.30 ± 0.49 GRW 0.85 ± 1.24 ARC% 91.47 ± 15.50 CRC% 66.20 12 M GRD 0.34 ± 0.49 GRW 0.95 ± 1.16 ARC% 90.48 ± 12.66 CRC% 57.75 24 M GRD 0.37 ± 0.55 GRW 0.98 ± 1.21 ARC% 89.90 ± 13.36 CRC% 57.75 Test (A-CTG + CAF) 6 M GRD 0.21 ± 0.37 GRW 0.69 ± 1.14 ARC% 93.22 ± 12.24 CRC% 71.43 12 M GRD 0.22 ± 0.36 GRW 0.73 ± 1.13 ARC% 92.64 ± 12.29 CRC% 68.83 24 M GRD 0.27 ± 0.44 GRW 0.80 ± 1.21 ARC% 91.76 ± 13.29 CRC% 67.53	Attached Gingiva AG Control (NA-CTG + CAF) Baseline 1.51 ± 1.78 6 M 3.93 ± 1.33 12 M 3.91 ± 1.29 24 M 3.87 ± 1.33 Test (A-CTG + CAF) Baseline 1.33 ± 1.41 6 M 3.73 ± 1.00 12 M 3.70 ± 0.94 24 M 3.69 ± 0.98	Not mentioned	6, 12, and 24 months
Bakhishov et al. (2021) [19]	modified Tunnel + CTG	-palate -DGG with intra-oral de-epithelialization -SCTG single-incision SI	DGG thickness 1.40 ± 0.11 width 20.42 ± 3.53 height 4.67 ± 0.49 SCTG thickness 1.37 ± 0.11 width 17.57 ± 2.38 height 4.64 ± 0.50	DGG RD 2.63 ± 0.89 RW 2.95 ± 0.87 SCTG RD 3.0 ± 1.19 RW 3.45 ± 0.68	DGG RD 6 M 0.52 ± 0.73 12 M 0.27 ± 0.54 RW 6 M 0.80 ± 0.99 12 M 0.77 ± 1.16 MRC% 6 M 84.72 ± 19.72 12 M 91.72 ± 16.59 CRC% 6 M 70.0 (21/30) 12 M 76.7 (23/30) SCTG RD 6 M 0.76 ± 0.91 12 M 0.68 ± 0.99 RW 6 M 1.19 ± 1.42 12 M 1.06 ± 1.41 MRC% 6 M 79.62 ± 22.51 12 M 83.16 ± 23.32 CRC% 6 M 48.4 (15/31) 12 M 61.3 (19/31)	DGG KTH baseline 2.70 ± 1.29 6 M 3.97 ± 1.03 12 M 4.20 ± 1.03 GT baseline 0.73 ± 0.19 6 M 1.54 ± 0.44 12 M 1.58 ± 0.54 SCTG KTH baseline 2.42 ± 1.18 6 M 3.48 ± 0.93 12 M 4.13 ± 0.96 GT baseline 0.74 ± 0.20 6 M 1.51 ± 0.38 12 M 1.57 ± 0.29 Histological results: Cellularity higher in SCTG, vascularity no difference, DGG showed partly epithelial remnants in superficial portions	Using VAS; No significant differences between groups in respect to postoperative pain, patients’ discomfort, sensitivity and the amount of systemic analgesic consumption	-Patient reported outcome 1, 2, 3, 7, 14 and 28 days. -Clinical outcome 6 and 12 months.
Amin et al. (2018) [2]	-CAF + CTG group (20 sites): split thickness flap. -FGG group (20 site): split-thickness flap with butt joint horizontal incision	palate and tuberosity -both harvested as epithelialized grafts via double blade scalpel handle -CTG de-epithailize extraorally flap	-Thickness 1.5 mm for all grafts harvested —For CTG cases, thickness of 1.0 mm was maintained after de-epithelialization.	not mentioned	mean RC% at 8 W (for 10 CAF and CTG pt): -Tuberosity 67 ± 12% -Palate 62 ± 13%	Mean GT (8 W): -Tuberosity CAF + CTG 2.9 ± 0.5 mm FGG 2.7 ± 0.7 mm -Palate CAF + CTG 2.3 ± 0.6 mm FGG 2.1 ± 0.7 mm Mean gingival thickness of the healed tuberosity grafts was greater than of the palatal grafts	mean pain score (10-point scale) at 2 W: -Tuberosity 2.6 ± 2.2 -Palate 5.9 ± 2.7	2, 4, and 8 weeks
Bednarz et al. (2016) [20]	MCAT modified coronally advanced tunnel	palate—SI harvesting technique	not mentioned	mm CTG RD0 2.50 ± 1.01 RW0 3.20 ± 0.99 mm FL RD0 2.19 ± 0.15 RW0 3.23 ± 1.09	CTG RD3 0.25 ± 0.44 RD6 0.13 ± 0.33 RW3 0.40 ± 0.81 RW6 0.28 ± 0.75 %ARC3 94.28 ± 0.11 %ARC6 95.77 ± 0.11 %CRC3 90.00 ± 0.18 %CRC6 94.87 ± 0.14 FL RD3 0.06 ± 0.24 RD6 0.13 ± 0.47 RW3 0.21 ± 0.92 RW6 0.35 ± 1.18 %ARC3 97.99 ± 0.09 %ARC6 94.21 ± 0.20 %CRC3 97.34 ± 0.11 %CRC6 94.24 ± 0.20	mm CTG HKT0 2.13 ± 1.47 HKT3 3.06 ± 1.83 HKT6 3.09 ± 0.95 mm FL HKT0 2.61 ± 1.36 HKT3 3.09 ± 1.03 HKT6 2.86 ± 1.60	Not mentioned	3 and 6 months
Pandit et al. (2016) [21]	full mucoperiosteal flap using horizontal and vertical incisions following Langer and Langer technique	palate—trap door technique: -group I Unigraft knife method. -group II Langer and Langer)	Thickness: -Unigraft knife 1.5 mm -Langer and Langer was not specified	mean Rec0: grp I 3.2 ± 1.42 grp II 3.6 ± 1.12	mean grp I Rec 3 1.6 ± 1.18 Rec 6 1.53 ± 1.14 mean grp II Rec 3 1.43 ± 1.4 Rec 6 1.33 ± 1.38	mean grp I KT0 2.8 ± 1.66 KT3 4.2 ± 1.74 KT6 4.27 ± 1.68 mean grp II KT0 2.4 ± 0.91 KT3 4.53 ± 1.25 KT6 4.67 ± 1.18	Using Visual analog scale (VAS) at 1-week: both groups exhibited with no significant difference Using Verbal rating scale (5-points VRS) at 1-week: group I had moderate intensity pain; group had II dull pain	1, 4, 12 weeks, 3, 6 months
Aroca et al. (2013) [22]	MCAT + CTG or CM	Palate —either modified distal wedge or SI	-CTG thickness 1–1.5 mm -CM width 5 mm	mean control GRD 1.8 ± 0.5 GRW 3.8 ± 0.9 mean test GRD 1.9 ± 0.6 GRW 3.8 ± 0.8	mean control GRD12 0.2 ± 0.3 GRW12 0.5 ± 1.0 %RC12 90 ± 18 mean test GRD12 0.6 ± 0.5 GRW12 1.4 ± 1.2 %RC12 71 ± 21	mean control KTW0 2.0 ± 0.7 KTW12 2.7 ± 0.8 GT0 0.8 ± 0.3 GT12 1.3 ± 0.4 mean test KTW0 2.1 ± 0.9 KTW12 2.4 ± 0.7 GT0 0.8 ± 0.2 GT12 1.0 ± 0.3	using VAS at 12 M—patient complaint: control 12.8 ± 7.5 test 7.3 ± 3.4 -patient satisfaction: control 90.6 ± 7.9 test 92.9 ± 8.4 -The number of 100% satisfaction was higher in the test group, but not statistically significant	6 and 12 months
Lafzi et al. (2007) [23]	CAF + CTG	palate—horizontal incision with vertical incision mesially	Thickness 0.9 mm	mean P-teeth RD 4.46 ± 0.48 RW 3.00 ± 0.65 mean P-flap RD 4.56 ± 0.56 RW 3.06 ± 0.49	mean P-teeth RD 0.78 ± 0.99 RW 0.31 ± 0.37 CRC 50% of the cases %RC 81.77 mean P-flap RD 1.31 ± 1.16 RW 0.43 ± 0.41 CRC 37.5% of the cases %RC 72.39	P-teeth LKT0 1.37 ± 0.53 LKT3 2.62 ± 0.64 P-flap LKT0 1.25 ± 0.49 LKT3 2.56 ± 0.56	using patient questioning: esthetic concerns were met in PRC above 70%, gradual reduction in PRC declined to 60%, sudden dissatisfaction in PRCs below 60%	1, 3 months
Wilson et al. (2005) [24]	CAF + CTG or HF-DDS	palate—no details	not mentioned	mean control RD 3.9 ± 0.88 RW 3.9 ± 0.88 mean test RD 3.7 ± 0.82 RW 4.2 ± 0.92	mean control test RD1 0.7 ± 0.68 1.4 ± 0.70 RD3 1.4 ± 0.97 1.9 ± 0.57 RD6 1.4 ± 1.30 1.6 ± 0.92 RW6 2.4 ± 2.07 3.4 ± 1.77 %RC1 83.7 ± 15.3 62.8 ± 17.6 %RC3 64.2 ± 23.8 47.0 ± 20.3 %RC6 64.4 ± 31.9 56.7 ± 27.8	mean control KT0 1.9 ± 0.88 KT6 2.1 ± 0.84 mean test KT0 1.9 ± 0.88 KT6 2.1 ± 0.64	questionnaire was used for discomfort and satisfaction. Patient satisfaction in regard to tissue contours and color match was similar regardless of the graft material used.	1 week after surgery, and at months 1, 3, and 6
Harris (1997) [25]	partial thickness double pedicle graft technique	palate—free gingival graft knife method or parallel incisions method	size mm^2^—FGGK 85.9 ± 23.6—PI 86.9 ± 23.1	mean FGGK RD 3.5 ± 0.8 RW 3.0 ± 0.6 mean PI RD 3.2 ± 0.9 RW 4.3 ± 2.0	mean12 W FGGK PI RD 0.1 ± 0.2 0.1 ± 0.3 RW 0.2 ± 0.5 0.2 ± 0.8 RC% 98.3 ± 4.2 98.7 ± 5 CRC% 84.2 93.3	mean FGGK KTW0 1.5 ± 1.4 KTW12 5.2 ± 1.6 mean PI KTW0 1.4 ± 1.2 KTW12 5.0 ± 1.2	-Patients were asked to rate discomfort level, and grouped as: no pain or minimal pain, or greater than minimal pain. -PI produced less postoperative discomfort compared to the FGGK method.	1, 2, 4, 8, 12 weeks postoperative
Wennstrom and Zucchelli (1996) [26]	CAF with/out CTG	palate—trap door technique	Thickness 1.5 to 2 mm	mean RD: control 4.1 ± 0.9 test 4.0 ± 1.0	mean control test RD 6 M 0.2 ± 0.4 0.2 ± 0.3 RD 12 M 0.1. ± 0.3 0.1 ± 0.2 RD 24 M 0.2 ± 0.3 0.1 ± 0.2 %RC 6 M 96.4 ± 6.5 96.1 ± 6.7 %RC 12 M 97.7 ± 5.2 98.7 ± 3.3 %RC2 4 M 97.1 ± 6.2 98.9 ± 3.1 %CRC 6 M 74 72 %CRC 12 M 83 86 %CRC 24 M 80 88	mean GH control baseline 1.1 ± 0.5 6 M 1.5 ± 0.5 12 M 2.3 ± 0.6 24 M 2.2 ± 0.6 mean GH test baseline 0.9 ± 0.5 6 M 3.5 ± 0.6 12 M 3.7 ± 0.7 24 M 3.7 ± 0.6	not mentioned	6, 12, 24 months

Abbreviations: %RC: percentage of root coverage; %CRC: percentage of complete root coverage; RD: recession dimension; MCAF: multiple coronally advanced flap; MCAF + CTG: multiple coronally advanced flap plus connective tissue graft; PFP: primary flap position; RecRed: recession reduction; ∆KTW: keratinized tissue width changes from baseline; ∆MTT: marginal tissue thickness changes from baseline; LKT: length of keratinized tissue; CAF: coronally advanced flap; CTG: connective tissue graft; MCAT: modified coronally advanced tunnel; CM: collagen matrix; HF-DDS: human fibroblast-derived dermal substitute, VAS: visual analog scale.

## Data Availability

All supporting data are included in the review. The raw data are available from the corresponding author upon request.

## Supplementary Materials

The following supporting information can be downloaded at: https://www.mdpi.com/article/10.3390/dj13120563/s1, Table S1: PRISMA 2020 Checklist.
